# Laparoscopic Gastrectomy for Cancer: Cut Down Complications to Unveil Positive Results of Minimally Invasive Approach

**DOI:** 10.3389/fonc.2022.854408

**Published:** 2022-03-04

**Authors:** Milos Bjelovic, Milan Veselinovic, Dragan Gunjic, Zoran Bukumiric, Tamara Babic, Radmila Vlajic, Dario Potkonjak

**Affiliations:** ^1^ Department for Minimally Invasive Upper Digestive Surgery, Hospital for Digestive Surgery, Clinical Center of Serbia, Belgrade, Serbia; ^2^ School of Medicine, University of Belgrade, Belgrade, Serbia; ^3^ Institute for Medical Statistics, Belgrade, Serbia

**Keywords:** gastrectomy, advanced gastric cancer (AGC), laparoscopic gastrectomy (LG), minimally invasive surgery, postoperative complications

## Abstract

Several randomized controlled trials and meta-analyses have confirmed the advantages of laparoscopic surgery in early gastric cancer, and there are indications that this may also apply in advanced distal gastric cancer. The study objective was to evaluate the safety and effectiveness of laparoscopic gastrectomy (LG), in comparison to open gastrectomy (OG), in the management of locally advanced gastric cancer. The single-center, case–control study included 204 patients, in conveyance sampling, who underwent radical gastrectomy for locally advanced gastric cancer. Out of 204 patients, 102 underwent LG, and 102 patients underwent OG. The primary endpoints were safety endpoints, i.e., complication rates, reoperation rates, and 30-day mortality rates. The secondary endpoints were efficacy endpoints, including perioperative characteristics and oncological outcomes. Even though the overall complication rate was higher in the OG group compared to the LG group (30.4% and 19.6%, respectively), the difference between groups did not reach statistical significance (p = 0.075). No significant difference was identified in reoperation rates and 30-day mortality rates. Time spent in the intensive care unit (ICU) and overall hospital stay were shorter in the LG group compared to the OG group (p < 0.001). Although the number of retrieved lymph nodes is oncologically adequate in both groups, the median number is higher in the OG group (35 vs. 29; p = 0.024). Resection margins came out to be negative in 92% of patients in the LG group and 73.1% in the OG group (p < 0.001). The study demonstrated statistically longer survival rates for the patients in the laparoscopic group, which particularly applies to patients in the most prevalent, third stage of the disease. When patients with the Clavien–Dindo grade ≥II were excluded from the survival analysis, further divergence of survival curves was observed. In conclusion, LG can be safely performed in patients with locally advanced gastric cancer and accomplish the oncological standard with short ICU and overall hospital stay. Since postoperative complications could affect overall treatment results and diminish and blur the positive effect of the minimally invasive approach, further clinical investigations should be focused on the patients with no surgical complications and on clinical practice to cut down the prevalence of complications.

## Introduction

When Erich Muhe and Phillipe Mouret first described laparoscopic cholecystectomy in 1985 and 1987, respectively, no one believed that large and demanding surgical procedures would be treated the same way in the future. But back in 1993, Juan Santiago Azagra performed the first laparoscopic-assisted total gastrectomy for gastric cancer.

In 1994, Kitano performed the first laparoscopic-assisted distal gastrectomy with a modified D1 lymph node dissection for the treatment of early gastric cancer, with a high risk of lymph node metastasis. Yasuhiro Kodera et al. heralded a whole new perspective for laparoscopic gastrectomy (LG) in 2010. They performed a meta-analysis, enrolling 6 randomized controlled trials and 666 patients, and they concluded that laparoscopic surgery with D2 lymphadenectomy for early gastric cancer is feasible and safe and adheres to the oncological principles (1).

Later on, several randomized controlled trials and meta-analyses have confirmed the advantages of laparoscopic surgery in early gastric cancer, and there are indications that this may also apply in advanced distal gastric cancer (1–3). However, in Western countries, the majority of patients still present with advanced stages of the disease. Locally advanced tumors require a more technically demanding procedure, especially in the case of total gastrectomy with intracorporeal esophagojejunal anastomosis. Nevertheless, laparoscopic surgery for gastric cancer has increased in popularity during the last two decades, in both the East and the West (4, 5). In addition, recent European-based studies found treatment results comparable with their Asian counterpart (6–8).

The study objective was to evaluate the safety and effectiveness of LG, in comparison to open gastrectomy (OG), in the management of locally advanced gastric cancer.

## Materials and Methods

The single-center, case–control study included 204 patients, in the convenience sampling, who underwent gastrectomy with a curative intention for locally advanced gastric cancer, between March 2013 and May 2021. Out of 204 patients, 102 underwent LG, and 102 patients underwent OG. Perioperative and postoperative data for the patients treated with LG were collected from a prospectively developed database. The OG group was a historical cohort. The study was reviewed and approved by the Clinical Centre of Serbia Institutional Review Board (decision number 187/15 dated October 20, 2016).

Through strategic change management, over the observed period, we have gradually increased the proportion of patients operated using the laparoscopic approach and decreased the proportion of patients operated using the open approach. Preoperative data did not influence the operative approach. Subsequent comparative analysis of the preoperative data did not indicate selection bias or potential confounding.

Inclusion criteria were as follows:

• patient’s age ≥18 and ≤80 years

• patients able to undergo general anesthesia and major surgery and are suitable laparoscopic surgical candidates

• patients who provided written informed consent after being informed of the study procedure and risks prior to any study-related events

• patients with documented locally advanced gastric cancer

Patients with other synchronous or metachronous neoplasms, preoperatively confirmed metastatic disease, histology other than adenocarcinoma, and poor general status with severe comorbidities were excluded from the study.

### Study Procedures

The study plan included a preoperative/baseline visit, a surgical procedure phase with hospital stay until discharge, and follow-up visits.

At the preoperative/baseline visit, the eligibility of subjects to receive treatment was determined. Before surgery, all patients underwent multidisciplinary team consultation with diagnostic and therapeutic workout according to the European Society for Medical Oncology (ESMO) recommendations (9).

Eligible subjects then undergo gastrectomy for cancer with curative intent. In the LG group, the positions of the patient and trocars were adopted from Luketich et al. (10); at the end of the procedure, the surgical specimen is placed in an extraction bag and removed from the abdomen through a 5-cm-long Pfannenstiel incision. A standard approach included omentectomy, D2 lymph node dissection, and total or subtotal gastrectomy, according to the criteria of the Japanese Gastric Cancer Association (JGCA) (9). Standard reconstruction after total gastrectomy was circular stapled esophagojejunal anastomosis utilizing double stapling technique with transabdominally inserted anvil (reverse-penetrating technique). In the laparoscopic approach, the insertion site of the stapler is in the left upper abdomen (11). The continuity of the digestive tube, in the patients with subtotal gastrectomy, was provided by forming retrocolic, inframesocolic hand-sewn gastro-jejunal anastomosis (Billroth II reconstruction–Finsterer-Hofmeister modification).

All patients underwent antibiotics and thromboembolic prophylaxis, as well as early mobilization after surgery. Control barium radiography was performed routinely on the fifth postoperative day after total gastrectomy, followed by a clear liquid diet. However, a control barium meal was not routinely performed after subtotal gastrectomy, and these patients began with the clear liquid diet on postoperative day three.

The specimen assessment was conducted through specified pathologists according to the 8th edition of the American Joint Committee on Cancer protocol from 2017. Localization and cell type of the tumor, as well as TNM status, were evaluated. Furthermore, the number of the harvested lymph nodes and the R status were assessed as key features of the oncological outcome of the methods in use (12).

After a discharge from the hospital, the first follow-up visits were at intervals of 3–4 months for the first year, six-monthly reviews for the second year, and annually thereafter.

### Study Endpoints

The primary endpoints were safety endpoints: non-inferiority of the LG group, compared to OE group, in the onset of

•total number and the most prevalent early postoperative complications (13, 14)

•complication classified according to the Clavien–Dindo classification (15)

•complications grade according to Comprehensive Complication Index (CCI) (16)

•reoperation rates and 30-day mortality rates

The secondary endpoints were efficacy endpoints, including perioperative characteristics and oncological outcomes:

•reduction in the LG group, compared to the OG group, in the intensive care unit (ICU) and overall hospital stay

•non-inferiority of the oncological outcomes of the LG group, compared to the OG group, based on the number of harvested lymph nodes, R status, and short-term survival.

### Statistical Analysis

Depending on the type of variables and the normality of the distribution, the data description is here presented as n (%), arithmetic means ± SD, or median (range, min–max). Among the methods for testing statistical hypotheses, the following were used: t-test, Mann–Whitney test, chi-square test, and Fisher’s test of exact probability. Logistic regression was used to analyze the relationship between binary outcomes and potential predictors. The Kaplan–Meier method was used to analyze the survival of patients with cancer, the log-rank test was used to assess the survival function of these patients depending on the type of surgery, and the Cox regression model with a 95% CI was used to find an independent predictor of death.

The data were censored for the following reasons: the respondent survived the entire follow-up period or was lost from the records. Statistical hypotheses were tested at the level of statistical significance (alpha level) of 0.05.

The results are presented in tables and graphs. All data were processed in the IBM SPSS Statistics 22 (SPSS Inc., Chicago, IL, USA) software package.

## Results

### Patient Characteristics, Surgical Procedures, and Tumor Characteristics

Two groups were homogenous in respect to average patients’ age, sex, body mass index (BMI), and American Society of Anesthesiologists physical status score (ASA score). They did not differ in respect to the type and duration of surgery, as well (**Table 1**).

**Table 1:** Patient characteristics, perioperative data, and surgical procedures.

	*Laparoscopic* (n = 102)	*Open* (n = 102)	p
Age^a^ (years)	63 (25–87)	64 (18–84)	0.595
Sex, males: n (%)	68 (66.7)	66 (64.7)	0.768
BMI^a^ ^,^ ^b^(kg/m^2^)	24.6 (16.8–46.6)	24.4 (16.2–35.4)	0.710
ASA^c^ score^a^			0.129
1: n (%)	40 (39.2)	30 (29.4)	
2: n (%)	42 (41.2)	46 (45.1)	
3: n (%)	20 (19.6)	26 (25.5)	
Extent of gastrectomy: n (%)			0.066
Total gastrectomy	52 (51.0)	65 (63.7)	
Subtotal gastrectomy	50 (49.0)	37 (36.3)	
Duration of surgery (min)^a^	290 (180–420)	270 (90–510)	0.058
Hospital stay (days)^a^	10 (4–27)	11 (6–26)	**<**0.**001**
ICU^d^ stay (days)^a^	1 (0–7)	1 (1–8)	**<**0.**001**

a Data shown represent median (range).

b BMI, body mass index.

c ASA, American Society of Anesthesiologists.

d ICU, intensive care unit.

In bold: statistically significant.

While tumor localization and stage of the disease have no significant difference between groups, the size of the tumor was statistically larger in the OG group (p = 0.023). The average tumor diameter in the LG group was 60 mm and in the OG group 70 mm. Observed groups did not differ in respect to the T stage (p = 0.107). T1 stage of the disease was found only in 12.1% of patients in the LG group and 15.2% in the OG group. In addition, more than three-quarters of all patients had ≥T3 tumor at the time of surgery (**Table 2**). Groups did not differ in respect to the N stage as well (p = 0.669). Negative lymph nodes, at the time of surgery, were observed in 32.3% of patients treated utilizing the minimally invasive (MI) approach and 30.3% of patients treated using the open approach.

**Table 2:** Histopathological findings.

	Laparoscopic	Open	p
Tumor localization			0.**022**
Upper third: n (%)	13 (12.7)	31 (30.4)	
Middle third: n (%)	33 (32.4)	26 (25.5)	
Lower third: n (%)	44 (43.1)	37 (36.3)	
Pangastric: n (%)	12 (11.8)	8 (7.8)	
Diameter of tumor (mm)^a^	60.0 (10–180)	70.0 (15–300)	0.**023**
R status			**<**0.**001**
R0 resection: n (%)	92 (92.0)	68 (73.1)	
R1 resection: n (%)	8 (8.0)	25 (26.9)	
T stage			0.107
T1: n (%)	12 (12.1)	15 (15.2)	
T2: n (%)	12 (12.1)	3 (3.0)	
T3: n (%)	40 (40.4)	33 (33.3)	
T4: n (%)	35 (35.4)	48 (48.5)	
N stage			0.669
N0: n (%)	32 (32.3)	30 (30.3)	
N1: n (%)	15 (15.2)	13 (13.1)	
N2: n (%)	18 (18.2)	20 (20.2)	
N3: n (%)	34 (34.3)	36 (36.4)	
Lymph nodes retrieved^a^	29 (15–74)	35 (15–81)	0.**024**
Positive lymph nodes^a^	3 (0–38)	4 (0–59)	0.487
AJCC^b^ pathological stage			0.259
I stage: n (%)	18 (18.2)	17 (17.2)	
II stage: n (%)	26 (26.3)	18 (18.2)	
III stage: n (%)	53 (53.5)	61 (61.6)	
IV stage: n (%)	2 (2.0)	3 (3.0)	

a Data shown represent median (range).

b American Joint Committee on Cancer, 8th edition.

In bold: statistically significant.

### Safety Endpoints (Prevalence of Significant Early Postoperative Complications)

Even though the overall complication rate was higher in the OG group, compared to the LG group (30.4% and 19.6% respectively), the difference between groups did not reach statistical significance (p = 0.075).

The statistics did not reach a significant difference even when each complication was analyzed individually. One of the most frequent complications was wound infection. Almost 3 times higher relative frequency of wound infections was identified in the OG group, compared to the LG group (8.8% vs. 2.9%). Nevertheless, due to low frequencies of outcomes of interest, no statistical significance was achieved (p = 0.074) (**Table 3**). The same is with other complications, including major postoperative pulmonary complications (MPPC) and anastomotic leakage. One patient in the OG group had type 2 leakage of the esophagojejunal anastomosis, and 2 patients in the OG group had MPPC. No such complications are observed in the LG group (**Table 3**).

**Table 3:** Postoperative complications.

	*Laparoscopic* (n = 102)	*Open* (n = 102)	p
Overall complications: n (%)	20 (19.6)	31 (30.4)	0.075
Wound infection: n (%)	3 (2.9)	9 (8.8)	0.074
Diarrhea: n (%)	3 (2.9)	9 (8.8)	0.074
Transient hepatic function damage: n (%)	1 (1.0)	0 (0)	1.000
Intraabdominal bleeding: n (%)	2 (2.0)	1 (1.0)	1.000
Intraabdominal collection: n (%)	0 (0)	1 (1.0)	1.000
Neurological: n (%)	2 (2.0)	1 (1.0)	1.000
Urinary tract infection: n (%)	2 (2.0)	2 (2.0)	1.000
Urinary retention: n (%)	1 (1.0)	1 (1.0)	1.000
Prolonged bowel paresis: n (%)	1 (1.0)	2 (2.0)	1.000
Leukopenia: n (%)	0 (0)	1 (1.0)	1.000
Thrombocytosis: n (%)	0 (0)	2 (2.0)	0.498
Fever: n (%)	2 (2.0)	7 (6.9)	0.170
Pneumonia: n (%)	1 (1.0)	2 (2.0)	1.000
Biliary fistula: n (%)	1 (1.0)	0 (0)	1.000
Ileus: n (%)	1 (1.0)	0 (0)	1.000
Anastomotic leak: n (%)	0 (0)	1 (1.0)	1.000
Respiratory failure: n (%)	0 (0)	2 (2.0)	0.498
Pulmonary embolism: n (%)	1 (1.0)	0 (0)	1.000
Clavien–Dindo: n (%)			0.067
0	82 (80.4)	71 (69.6)	
I	9 (8.8)	11 (10.8)	
II	8 (7.8)	16 (15.7)	
III	2 (2.0)	1 (1.0)	
IV	1 (1.0)	2 (2.0)	
V	0 (0)	1 (1.0)	
CCI^a^ ^,^ ^b^	3.6 (0–42.4)	6.7 (0–100)	0.060
MPPC^c^: n (%)	0 (0)	2 (2.0)	0.498
Reoperation: n (%)	2 (2.0)	1 (1.0)	1.000
30-day mortality: n (%)	0 (0)	1 (1)	1.000

a Data shown represent median (range).

b CCI, Comprehensive Complication Index.

c MPPC, major postoperative pulmonary complications.

By analyzing complications according to the Clavien–Dindo classification (CDC) and CCI, no statistically significant difference between the two groups was observed (**Table 3**). Complications classified as the Clavien–Dindo grade ≥II had 10.8% patients in the LG group and 19.7% in the OG group (p = 0.067). In addition, although 80.4% of patients in the LG group had a CCI score of 0, compared to 69.6% of patients in the OG group, no statistically significant difference between groups was achieved (p = 0.060).

Based on the analysis, no statistically significant difference was identified in reoperation rates and 30-day mortality rates (**Table 3**).

### Perioperative Characteristics

Time spent in the ICU was significantly shorter in the LG group with an average value of 1.0 compared to 1.5 days for the OG group (p < 0.001). A statistically significant difference is also observed in the length of hospital stay (10 vs. 11 days) (p < 0.001) (**Table 1**). In the group of laparoscopically treated patients, there were no conversions to open surgery.

### Oncological Outcomes

Although the number of retrieved lymph nodes is oncologically adequate in both groups, the median number is significantly higher in the OG group (35 vs. 29; p = 0.024). Resection margins came out to be negative in 92% of patients in the LG group and 73.1% in the OG group (p < 0.001). The vast majority of these patients in the OG group had a positive circumferential resection margin.

### Follow-Up

**Figure 1:**
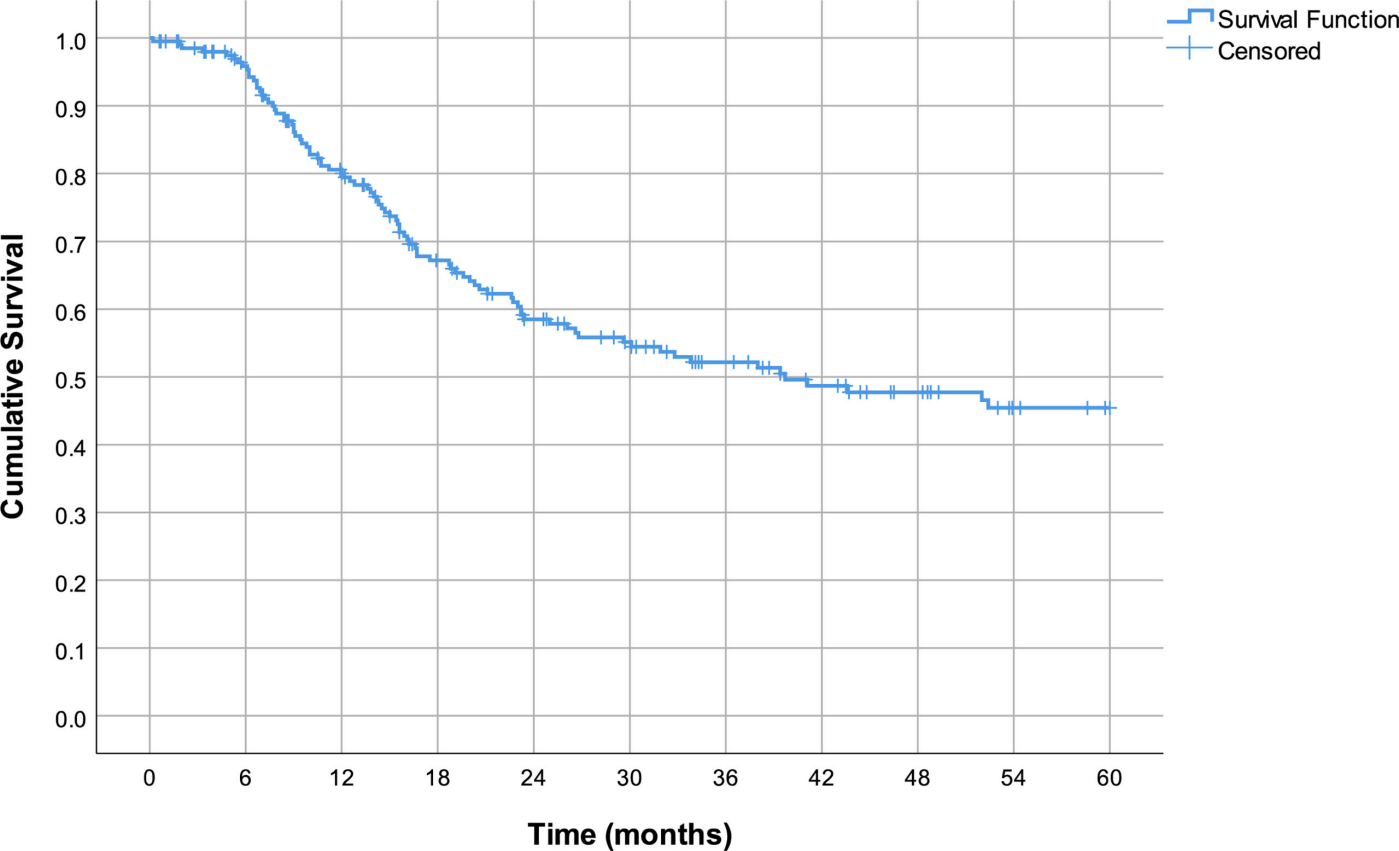
Estimated mean overall survival.

The estimated mean survival in all treated patients was 37.4 months (95% CI 34.0–40.8) (**Figure 1**). The estimated mean survival in the LG group was 41.8 months (95% CI 36.9–46.7), while in the OG group, it was 33.8 months (95% CI 29.2–38.4) (p = 0.018).

**Figure 2:**
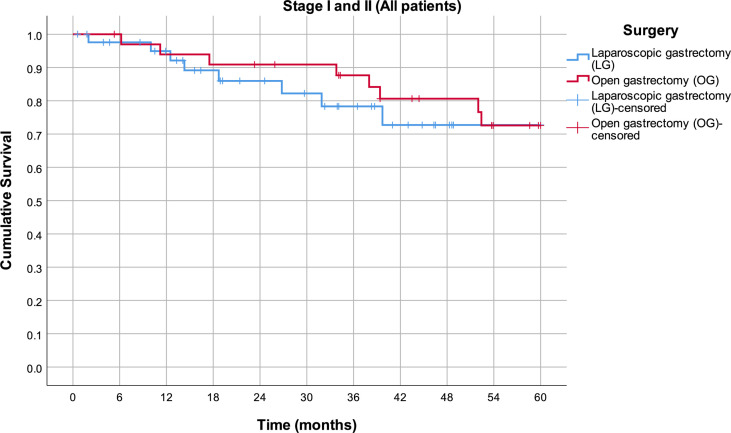
Estimated mean survival for the patients in stage I and II disease, in respect to the operative approach.

**Figure 3:**
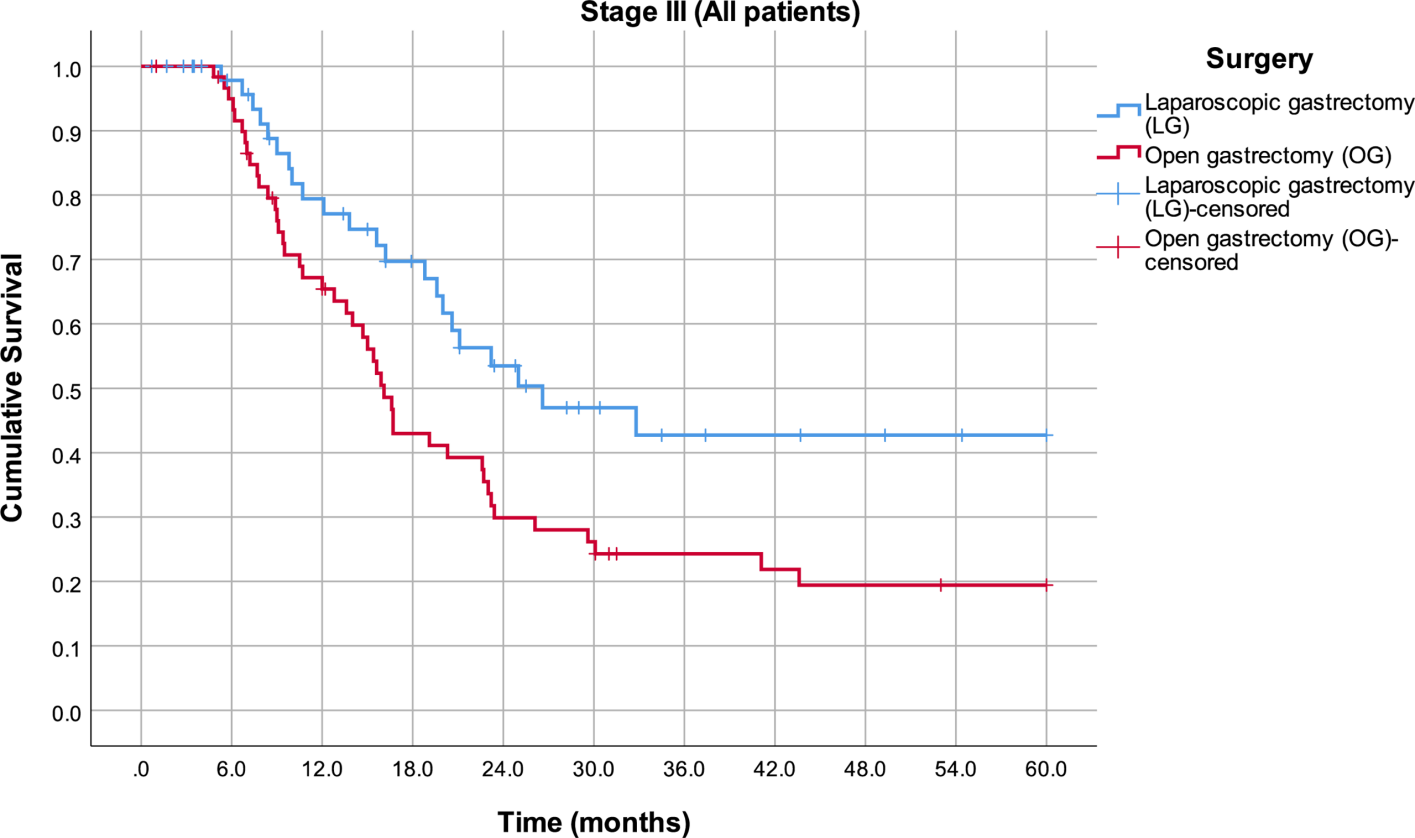
Estimated mean survival for the patients in stage III disease, in respect to the operative approach.

By analyzing mean survival rates only for the patients who had stage I and II disease, no statistical significance was found (p = 0.566): 49.8 months in the LG group (95% CI 43.6–56.0) and 52.6 months in the OG group (95% CI 47.5–57.8) (**Figure 2**). For the patients with stage III disease, the median survival rate in the LG group was 26.6 months (95% CI 12.8–40.4) and in the OG group 16.1 months (95% CI 14.4–17.8), which is statistically significant (p = 0.014) (**Figure 3**).

The multivariate Cox regression model included predictors of death after surgery, which were statistically significant in univariate regression models, at a significance level of 0.05 (**Table 4**). In this model, the variables associated with increased mortality hazard are presence of the surgical complications (Clavien–Dindo grade ≥II (B = 2.100; p = 0.001)) and higher stage of the disease (higher T stage (B = 0.394; p = 0.018), higher N stage (B = 0.384; p = 0.002), and metastatic disease (B = 1.768; p < 0.001)). Even though surgical access in the multivariate model did not reach statistical significance, the hazard ratio (HR) for open surgery compared to laparoscopy is 1.5.

**Table 4:** Multivariate Cox regression model.

	B	p	HR	95.0% CI for Exp(B)	
				Lower	Upper
Surgical access	0.403	0.095	1.50	0.933	2.402
Sex	0.340	0.220	0.71	0.414	1.225
Clavien–Dindo	2.100	**0.001**	8.17	2.254	29.586
Tumor diameter	0.001	0.815	1.00	0.994	1.007
R status	0.218	0.450	1.24	0.706	2.190
T stage	0.394	**0.018**	1.48	1.071	2.055
N stage	0.384	**0.002**	1.47	1.149	1.877
M stage	1.768	**<0.001**	5.86	2.178	15.750

HR, hazard ratio.

In bold: statistically significant.

**Figure 4:**
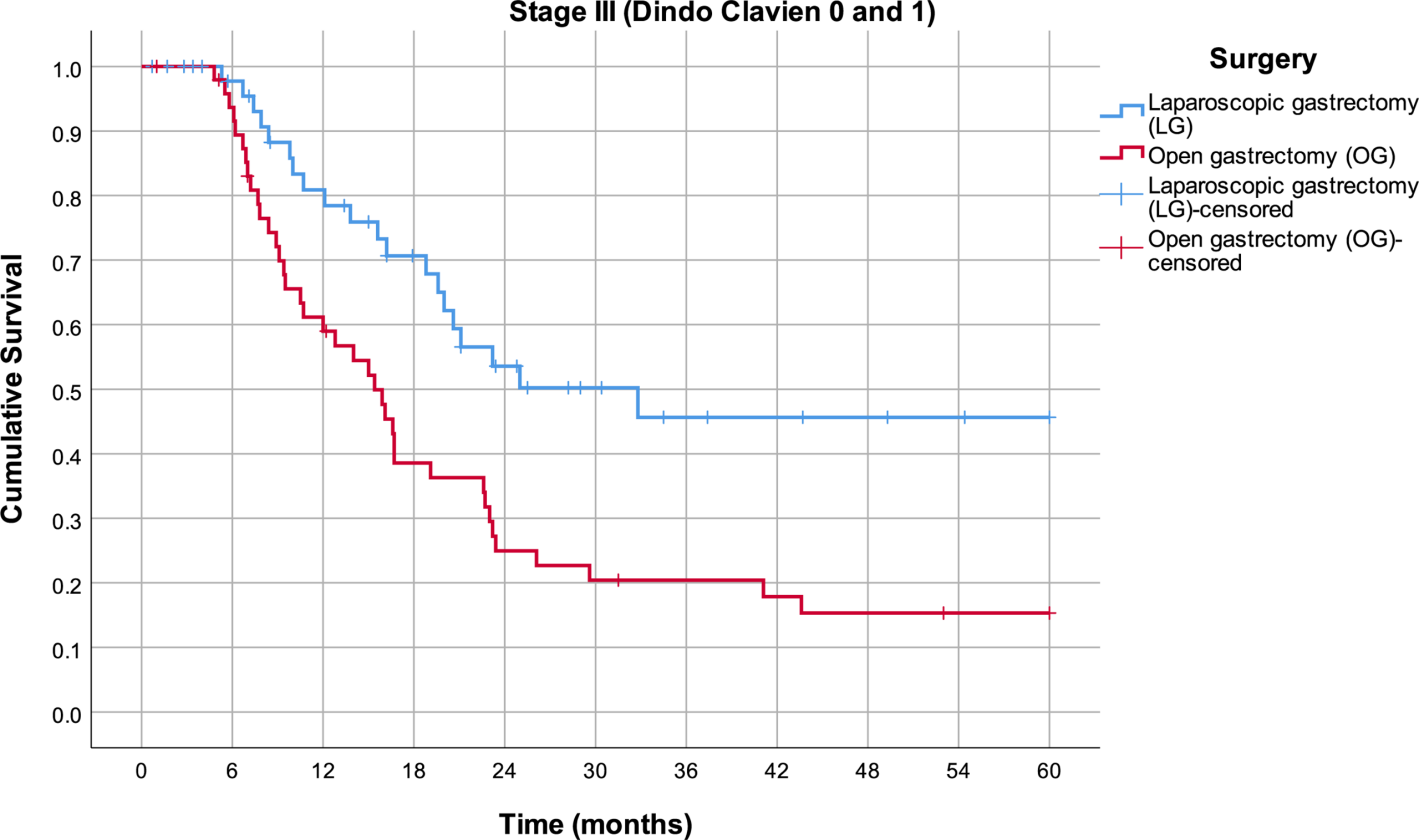
Estimated mean survival for the Clavien–Dindo group 0 and 1 patients in stage III disease, in respect to the operative approach.

By excluding patients who had complications grade ≥II according to the Clavien–Dindo classification, a further divergence of survival curves is observed (**Figure 4**). In the third stage of the disease, the mean survival rate in the LG group is 36.3 months (95% CI 29.0–43.6), while in the OG group, it is 22.0 months (95% CI 16.7–27.4) (p = 0.002).

## Discussion

Several randomized controlled trials and meta-analyses have confirmed the advantages of LG, compared to OG, in the treatment of early gastric cancer (1–3). However, in Western countries, the vast majority of patients still present with advanced stages of the disease and often with proximal tumor localization. Thus, despite that laparoscopic surgery has increased in popularity, uptake of the MI approach in the treatment of gastric cancer in Europe is relatively slow. Nevertheless, Hawerkamp et al. in 2016 and later on Chevallay, Bracale, and others demonstrated that European-based studies found that LG can be performed in Western European patients with locally advanced gastric cancer and meets the oncological standard with a short hospital stay when performed by trained surgeons (6–8).

This study analyzed the safety and efficacy of LG for locally advanced gastric cancer in a tertiary referral center in Serbia. In this single-center, case–control study, observed groups were homogenous in respect to patient demographic characteristics, type of surgery, tumor localization, and stage of the disease. All patients in this trial were initially diagnosed with locally advanced tumors. After neoadjuvant treatment, patients were operated on, and definitive histology was clarified according to the final histopathological findings (PH). In the final analysis, there was a subgroup of patients with T1 tumors. One of many possible explanations is that clinical TNM did not match ideally with the pathological TNM staging. Another could be that, to some point, regression of the tumor could be expected with neoadjuvant treatment, but the correlation between the clinical and pathological treatment response is weak. At the end of the day, some patients with T1 (especially T1b) tumors were node positive.

This study demonstrated evidently lower complication rates in the MI group, yet not reaching a statistically significant difference (19.6% vs. 30.4%; p = 0.075). The study by Van der Wielen et al. (17) analyzed the outcome differences between East and West in MI gastrectomy versus OG. They found that the overall complication rates for the LG group were 21.69% and for OG 30.80% in the Western studies. Differently, the rates in Asian studies showed complication rates of 12.23% in the LG group and 15.79% in the OG group. Recently published Western trials found results comparable with those of the Asian counterpart. To our knowledge, the lowest quoted overall complication rate, after LG for cancer, was 15.8% (18).

The most frequent complication, in our study, was wound infection. Although statistically not significant, it was 3 times more common in the OG group compared to the LG group (2.9% vs. 8.8%). One of the most fearsome complications is anastomotic leakage. There was one patient in the OG group with type 2 leakage of the esophagojejunal anastomosis, a rate that is comparable to that of most Western and Eastern studies (6, 19). No difference was observed in MPPC, reoperation, and 30-day mortality rates.

In most trials, the number of overall complications matches complication rates according to the Clavien–Dindo classification. However, Clavien–Dindo classification does not sum up all of the complications that occurred, but only the gravest. Thus, a limitation of this classification is that events of lesser severity may not be considered, leading to an underestimation of the true overall postoperative morbidity. The CCI has been shown to yield a substantial additional value to the Clavien–Dindo classification in patients with more than 1 complication.

Prevalence of complications defined as the Clavien–Dindo grade ≥II is almost two times lower in the LG group, compared to the OG group (10.8% and 19.7% respectively); nevertheless, the difference did not reach statistical significance (p = 0.067). Similar results have been achieved when the severity of complications, with grade according to the CCI, was analyzed. The mean CCI index was 3.6 in the LG group and 6.7 in the OG group, yet not reaching the level of statistical significance.

When we look beyond the percentage of specific complications, we can find that Tsukada et al. have reported that elevated levels of inflammatory mediators like cytokines could be the cause of complications following major cancer surgery (20). When the production of cytokines was evaluated in patients undergoing major cancer surgery, lower production of cytokines was noted in the group of patients treated by utilizing the MI approach, compared to the open approach. In order to measure the invasiveness of MI esophagectomy (MIE), it might be necessary to evaluate other parameters in addition to morbidity rates. Moreover, there is a possibility that the overall number of cases in our study was too small to reach statistical significance.

The mean duration of surgery for the LG group and OG group was 290 and 270 min, respectively, and is comparable to that in most Western series (6, 7, 17) and slightly longer than that in the Asian studies (17, 21). Introducing technically demanding procedure needs must not unduly prolong operations. The effects of the learning curve in our opinion were minimized with excessive experience in other advanced upper gastrointestinal laparoscopic and thoracoscopic procedures with the same surgical team performing all operations.

The duration of routine postoperative ICU stay/recovery and hospital stay, although unreliable as a criterion of outcome among centers, is a useful parameter of the severity of the postoperative course and complication within a single center, particularly when there are defined protocols and discharge policy (15). Nevertheless, in our study, the average ICU stay and length of hospital stay were significantly reduced in the LG group when compared to the OG group, suggesting an earlier recovery in the case of LG.

Regarding oncological outcomes, significantly more lymph nodes were retrieved in the OG group, compared to the LG group, even though the mean number of harvested lymph nodes in the LG group also met the criteria for adequate radical lymphadenectomy. Our results were more comparable to those reported in Eastern studies and somewhat better than in Western studies (17, 22). Data also revealed a higher R0 rate in the LG group (92% vs. 73.1%), which can be justified by a more advanced T category and mean tumor size in the open group, with the majority of R1 resections at circumferential resection margin.

Numerous Western and Eastern studies demonstrated that the long-term survival and recurrence rates of laparoscopic gastric cancer surgery are comparable to those of open surgery for the treatment of both early and advanced stage gastric cancer (23–26). Garbarino et al., comparing laparoscopic versus open distal gastrectomy for locally advanced gastric cancer, pointed out completely different survival results. Patients with N0 or stage IB-II had better survival after LG. On the contrary, N+ and stage III patients had no survival benefit due to the laparoscopic approach (27). Observing the late outcome, our study demonstrated statistically longer survival rates for the patients in the laparoscopic group, which particularly applies to patients in the most prevalent, third stage of the disease. However, the survival inferiority of the OG group is probably related to the more advanced tumor, rather than the operative approach itself. The analyzed groups did not differ statistically in respect to the T, N, and M status and tumor stage. Nevertheless, due to the small sample size, patients were classified into the single-stage III rather than stage IIIA, IIIB, or IIIC. Out of the total number of patients in the stage III disease, in the OG group, more than 27% were in stage IIIC, while in the laparoscopic group, there were only 18% in this most advanced III stage. This observation is further supported by the multivariate statistics where survival is influenced by the stage of the disease and the presence of the surgical complications, rather than the surgical approach. Nevertheless, in the multivariate model, HR for open surgery compared to laparoscopy is 1.5. In addition, a significant difference was observed in respect to the tumor size and R status, and possible oncological impact of the higher R1 status on the OG patients should not be underestimated.

To check to what extent postoperative complications influence the positive effects of the MI approach, all patients with the Clavien–Dindo grade ≥II were excluded from the survival analysis, and further divergence of survival curves was observed. That could mean that postoperative complications could adversely affect the positive effects of the MI approach when the total population of patients is analyzed.

Our study showed that postoperative complications could diminish and blur the positive effect of the laparoscopic approach. Thus, future research focused on the evaluation of the MI approach in major cancer surgery should be focused on the patients with no complications. In addition, clinical practice should be focused on complication prediction and prevention to reduce their clinical and oncological impact.

The study has several limitations. First, the sample size is relatively small and has limited power to compare low frequencies of outcomes of interest. Second, this is a case–control study with a historical cohort and is subjected to selection biases. The importance of the MI approach in the treatment of locally advanced gastric cancer should be further tested in future, single-institution, randomized controlled trials. We strongly suggest testing the benefit of the MI approach separately in the subpopulation of patients with no significant surgical complications.

## Conclusion

In conclusion, LG can be safely performed in patients with locally advanced gastric cancer and accomplish the oncological standard with short ICU and overall hospital stay, when performed by surgeons trained in gastric cancer and advanced laparoscopic surgery. Since postoperative complications could affect overall treatment results and diminish and blur the positive effect of the MI approach, further clinical investigations should be focused on the patients with no surgical complications and clinical practice to cut down the prevalence of complications.

## Data Availability Statement

The original contributions presented in the study are included in the article/supplementary material. Further inquiries can be directed to the corresponding author.

## Ethics Statement

The studies involving human participants were reviewed and approved by the Clinical Centre of Serbia Institutional Review Board (decision number 187/15 dated October 20, 2016). The patients/participants provided their written informed consent to participate in this study.

## Author Contributions

MB contributed to the conception and design of the study. MV organized the database. ZB performed the statistical analysis. MV wrote the first draft of the manuscript. DG, TB, RV, and DP wrote sections of the manuscript and participated in data collection. All authors contributed to manuscript revision and read and approved the submitted version.

## Conflict of Interest

The authors declare that the research was conducted in the absence of any commercial or financial relationships that could be construed as a potential conflict of interest.

## Publisher’s Note

All claims expressed in this article are solely those of the authors and do not necessarily represent those of their affiliated organizations, or those of the publisher, the editors and the reviewers. Any product that may be evaluated in this article, or claim that may be made by its manufacturer, is not guaranteed or endorsed by the publisher.
